# Hepatocyte-Specific Smad4 Deficiency Alleviates Liver Fibrosis via the p38/p65 Pathway

**DOI:** 10.3390/ijms231911696

**Published:** 2022-10-02

**Authors:** Miaomiao Wei, Xinlong Yan, Xin Xin, Haiqiang Chen, Lingling Hou, Jinhua Zhang

**Affiliations:** 1 The College of Life Science and Bioengineering, Beijing Jiaotong University, Beijing 100044, China; 2 Faculty of Environmental and Life Sciences, Beijing University of Technology, Beijing 100124, China

**Keywords:** hepatocyte, Smad4, hepatic stellate cells, liver fibrosis, CTGF

## Abstract

Liver fibrosis is a wound-healing response caused by the abnormal accumulation of extracellular matrix, which is produced by activated hepatic stellate cells (HSCs). Most studies have focused on the activated HSCs themselves in liver fibrosis, and whether hepatocytes can modulate the process of fibrosis is still unclear. Sma mothers against decapentaplegic homologue 4 (Smad4) is a key intracellular transcription mediator of transforming growth factor-β (TGF-β) during the development and progression of liver fibrosis. However, the role of hepatocyte Smad4 in the development of fibrosis is poorly elucidated. Here, to explore the functional role of hepatocyte Smad4 and the molecular mechanism in liver fibrosis, a CCl_4_-induced liver fibrosis model was established in mice with hepatocyte-specific Smad4 deletion (Smad4^Δhep^). We found that hepatocyte-specific Smad4 deficiency reduced liver inflammation and fibrosis, alleviated epithelial-mesenchymal transition, and inhibited hepatocyte proliferation and migration. Molecularly, Smad4 deletion in hepatocytes suppressed the expression of inhibitor of differentiation 1 (ID1) and the secretion of connective tissue growth factor (CTGF) of hepatocytes, which subsequently activated the p38 and p65 signaling pathways of HSCs in an epidermal growth factor receptor-dependent manner. Taken together, our results clearly demonstrate that the Smad4 expression in hepatocytes plays an important role in promoting liver fibrosis and could therefore be a promising target for future anti-fibrotic therapy.

## 1. Introduction

Liver fibrosis is a wound-healing response caused by the abnormal accumulation of extracellular matrix (ECM) in various chronic liver diseases, including viral hepatitis, alcoholic liver disease (ALD), nonalcoholic steatohepatitis (NASH), autoimmune liver disease (AILD), metabolic liver disease, and schistosomiasis infection [1]. If the fibrotic process is highly progressive, fibrosis can develop into cirrhosis, which accounts for approximately one million deaths per year worldwide, or even hepatocellular carcinoma (HCC) [2], which is the fourth leading cause of cancer-related deaths globally [3]. Therefore, it is important to understand the molecular mechanisms underlying liver fibrosis to improve the prevention and treatment of liver fibrosis and HCC.

In the process of liver fibrosis, ECM mainly comes from activated myofibroblasts [1]. Activated HSCs are the main source of myofibroblasts. Therefore, HSCs activation has been identified as a central driver of liver fibrosis by promoting ECM accumulation [4]. In normal liver, quiescent HSCs (qHSCs) reside in the space of Disse, where they store vitamins. However, persistent liver injury and subsequent inflammatory responses upregulate multiple factors, including cytokines, chemokines, reactive oxygen species (ROS), and iron, which can stimulate HSCs activation and proliferation. Unlike qHSCs, activated HSCs (aHSCs) express alpha smooth muscle actin (α-SMA) and secret ECM components [4,5]. Thus, inhibiting HSCs activation may be an effective strategy for anti-fibrotic therapy.

In addition to activated HSCs, myofibroblasts in liver fibrosis may originate from epithelial cells including hepatocytes and bile duct cells [6]. Zeisberg et al. demonstrated that hepatocytes can participate in the process of liver fibrosis through epithelial mesenchymal transformation (EMT) in a CCl_4_-treated transgenic mouse model [7]. Furthermore, studies have shown that EMT from hepatocytes to myofibroblasts is considered to be a key process in liver fibrosis [6,8].

The liver possesses a rich cellular environment that is mainly composed of parenchymal cells and nonparenchymal cells, which jointly regulate fibrosis formation and regression. Hepatocytes, the dominant parenchymal cell type in the liver, actively coordinate the profibrogenic response and play a key role in the process of liver fibrosis [9]. Damaged hepatocytes are “promoters” that participate in the initiation and persistence of HSCs activation by releasing various compounds, such as ROS, cytokines, chemokines, and growth factors [10]. In addition, pro-apoptotic signals induce hepatocyte apoptosis that closely correlates with liver inflammation and HSCs activation. HSCs and Kupffer cells phagocytose hepatocyte-derived apoptotic bodies, thereby enhancing the expression of profibrogenic genes and death ligands, such as FasL [4,11].

The transforming growth factor (TGF-β) superfamily plays an important role in the development of liver fibrosis, and intracellular TGF-β signal transduction is mediated by Smad proteins [12]. The eight members of the mammalian Smad family are divided into distinct classes: receptor-regulated Smad (Smad1, 2, 3, 5, and 8), common Smad (Smad4), and inhibitory Smad (Smad6 and Smad7) [12,13]. Smad4 is a core mediator of the TGF-β signaling pathway, which interacts with the MAPK, PI3K/AKT, NF-κB, and Wnt/β-catenin signaling pathways. In addition, Smad4 plays a pivotal role in the switch of TGF-β function in liver fibrosis and inflammation [14]. In chronic hepatitis C, liver tissues display higher Smad4 immunopositivity. The expression level of Smad4 in hepatocytes of advanced liver fibrosis stage was higher than that in hepatocytes of early liver fibrosis stage [15]. Similarly, Qin et al. found that hepatocyte-specific Smad4 deletion inhibited lipogenesis and alleviated inflammation and apoptosis in NASH [16]. Yang et al. also confirmed that *Smad4* deficiency in hepatocytes weakened spontaneous liver injury, inflammation, fibrosis, and HCC in mice with hepatocyte-specific *TAK1* deletion [17]. Although Smad4 deletion in LX-2 cells led to the decreased expression of fibrotic genes, including collagen type I (Col1a1), α-SMA, TGF-β, and the tissue inhibitor of metalloproteinases 1 (TIMP1) [18], Wang et al. found no significant defects in Smad4 mutant mice before 8 months of age, and only some fibrosis and neutrophil accumulation in the livers over 8 months of age [19]. The malignant progression of liver fibrosis can lead to the occurrence of HCC. Some evidence demonstrated that knockdown of Smad4 inhibited cell migration and invasion in HCC [20]. Although many studies have investigated the role of Smad4 in liver diseases, the function and mechanism of hepatocyte Smad4 during early liver fibrosis remains unclear.

In this study, we established a mouse model of hepatocyte-specific Smad4 deletion to explore the functional role and molecular mechanism of Smad4 in liver fibrosis. Our results showed that Smad4 deletion in hepatocytes decreased CCl_4_-induced liver fibrosis by regulating the expression of inhibitor of differentiation 1 (ID1) and the secretion of connective tissue growth factor (CTGF) in hepatocytes. Furthermore, hepatocyte-specific Smad4 deletion promoted HSCs activation via the p38/p65 pathway in an epidermal growth factor receptor (EGFR)-dependent manner. Together, our findings demonstrated that Smad4 expression in hepatocytes can promote fibrosis during the pathogenesis of early hepatic fibrosis.

## 2. Results

### 2.1. Smad4 Expression Is Upregulated in Hepatocytes during Liver Fibrosis

**Figure 1:**
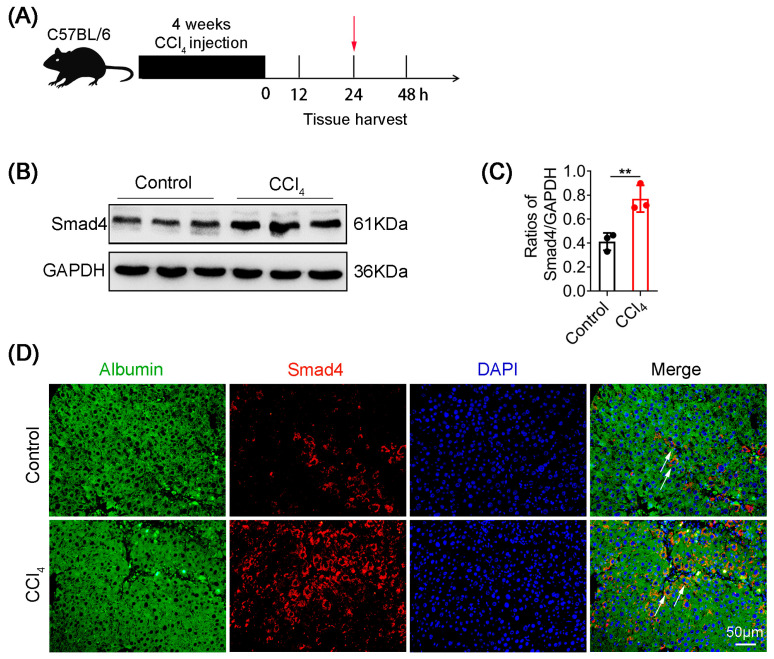
Smad4 expression is upregulated in hepatocytes during liver fibrosis. Groups of C57BL/6 mice were treated with CCl_4_ for 4 weeks to establish a liver fibrosis model (*n* = 6 per group). Data represent at least three independent experiments. (**A**) Schematic representation of CCl_4_-induced liver fibrosis. (**B**,**C**) Western blotting analysis of Smad4 protein levels in liver fibrosis tissues. Smad4 expression was normalized to the control GAPDH. (**D**) Double staining of albumin (green) and Smad4 (red) in liver fibrosis tissues (scale bars: 50 μm). Arrowheads indicated the double-positive cells. ** *p* < 0.01.

To investigate the functional role of Smad4 in liver fibrosis, C57BL/6 mice were administered CCl_4_ to establish liver fibrosis model, and liver tissues were harvested at 24 h after the last CCl_4_ injection (Figure 1A). Western blotting analysis of Smad4 expression in liver tissues revealed that Smad4 expression was significantly upregulated in mice with liver fibrosis compared with control mice (Figure 1B,C). Consistently, double immunofluorescence staining further indicated that Smad4 was highly expressed in hepatocytes in liver fibrosis tissues (Figure 1D). Collectively, these results demonstrated that Smad4 expression was significantly enhanced in hepatocytes during the progression of liver fibrosis.

### 2.2. Hepatocyte-Specific Smad4 Deficiency Attenuates Liver Fibrosis

**Figure 2:**
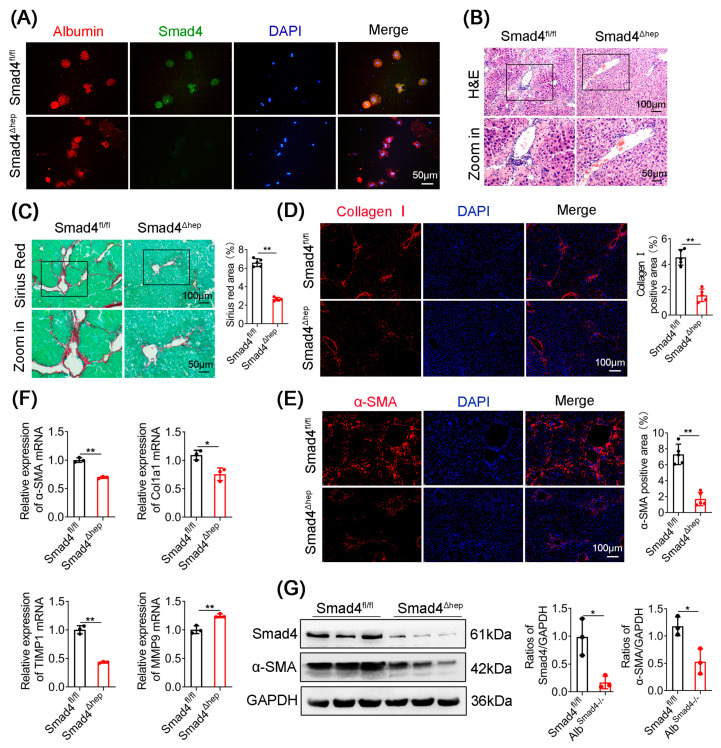
Hepatocyte-specific Smad4 deficiency attenuates liver fibrosis. Smad4^fl/fl^ and Smad4^Δhep^ mice were treated with CCl_4_ for 4 weeks to establish a liver fibrosis model (*n* = 6 per group). (**A**) Double staining of Albumin (red) and Smad4 (green) in primary hepatocytes (scale bars: 50 μm). (**B**) H&E staining of fibrotic liver tissues (scale bars: 100 μm, zoom in: 50 μm). (**C**) Sirius Red staining of fibrotic liver tissues (scale bars: 100 μm, zoom in: 50 μm), quantification of stained areas, and statistical analysis. (**D**,**E**) Immunofluorescence detection of collagen I and α-SMA in fibrotic liver tissues (scale bars: 100 μm), quantification of stained areas, and statistical analysis. (**F**) The mRNA levels of α-SMA, Col1a1, TIMP1, and MMP9 in fibrotic liver tissues were measured using qRT-PCR analysis. (**G**) Western blotting analysis of Smad4 and α-SMA protein levels in fibrotic liver tissues. Protein density was quantified using densitometry. α-SMA and Smad4 levels were normalized to GAPDH. * *p* < 0.05, ** *p* < 0.01.

To identify the role of hepatocyte Smad4 in liver fibrosis, transgenic mice expressing Cre recombinase from the Albumin promoter were crossed with Smad4^fl/fl^ mice to achieve hepatocyte-specific Smad4 ablation (Smad4^Δhep^). Smad4^Δhep^ mice were born at the expected Mendelian ratio. The Smad4^fl/fl^ littermates were used as control mice. The specific knockout of Smad4 in hepatocytes from Smad4^Δhep^ mice was confirmed by double immunofluorescence staining (Figure 2A and Figure S1A). H&E and Sirius Red staining demonstrated that inflammatory cell infiltration and collagen deposition decreased in the liver tissues of CCl_4_-treated Smad4^Δhep^ mice compared with those in Smad4^fl/fl^ mice (Figure 2B,C and Figure S1B, C). Consistently, lower collagen I expression was observed in CCl_4_-treated Smad4^Δhep^ mice livers (Figure 2D). Moreover, the infiltration of F4/80^+^ macrophages, CD11b^+^ macrophages, and Gr1^+^ neutrophils were markedly lower in liver tissues from Smad4^Δhep^ mice compared with those from Smad4^fl/fl^ mice following CCl_4_ treatment (Figure S2).

Immunofluorescence staining and qRT-PCR results indicated that hepatocyte-specific Smad4 deletion downregulated the expression of α-SMA (Figure 2E,F), suggesting that Smad4 deficiency in hepatocytes might alleviate the activation of HSCs. In addition, Smad4 deficiency in hepatocytes affected the expression of fibrosis-related genes, dramatically reducing the expression of Col1a1 and TIMP1 while increasing the expression of matrix metalloproteinase 9 (MMP9) at the mRNA level (Figure 2F). Consistently, Western blotting results indicated that α-SMA expression was lower in the liver tissues of CCl_4_-treated Smad4^Δhep^ mice than that in control mice (Figure 2G). Taken together, these findings suggested that Smad4 knockout in hepatocytes attenuated CCl_4_-induced liver fibrosis.

### 2.3. Hepatocyte-Specific Smad4 Deficiency Reduces Cell Proliferation and EMT

**Figure 3:**
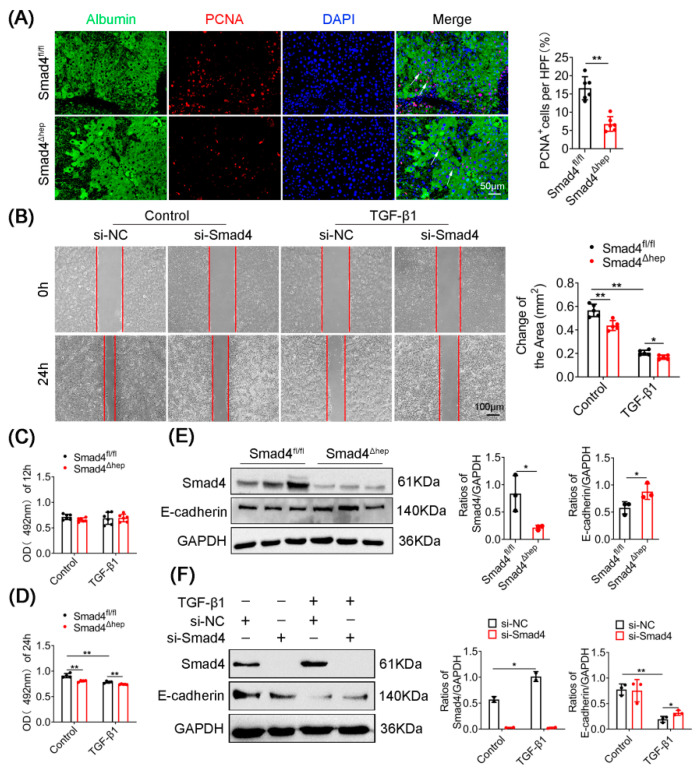
Hepatocyte-specific Smad4 deficiency reduces cell proliferation and EMT. Smad4^fl/fl^ and Smad4^Δhep^ mice were treated with CCl_4_ for 4 weeks to establish a liver fibrosis model. AML-12 cells were transfected with control siRNA or Smad4 siRNA. (**A**) Double staining of Albumin (green) and PCNA (red) in fibrotic liver tissues (scale bars: 50 μm) and statistical analysis. Arrowheads indicated the double-positive cells. (**B**) Representative photographs of wound-healing assay and statistical analysis. AML-12 cells were scratched using pipet tips and treated with TGF-β1 (5 ng/mL) for 24 h. The migration ability of AML-12 cells was evaluated. (**C**,**D**) AML-12 cells after Smad4 deletion were treated with TGF-β1 (5 ng/mL) for 12 h or 24 h. The MTT assays showed that the proliferation ability of AML-12 cells. (**E**) Smad4 and E-cadherin expression in fibrotic liver tissues of CCl_4_-treated Smad4^Δhep^ mice were analyzed by Western blotting. Smad4 and E-cadherin were normalized to GAPDH. (**F**) Smad4 in AML-12 cells were knocked down by siRNA and then treated with TGF-β1 (5 ng/mL) for 24 h; Smad4 and E-cadherin expressions were analyzed by Western blotting and normalized to GAPDH. * *p* < 0.05, ** *p* < 0.01.

To confirm whether Smad4 affects the proliferation of hepatocytes, PCNA and Albumin in the liver tissues of Smad4^Δhep^ and Smad4^fl/fl^ mice were determined by immunofluorescence double staining. The results showed that the proliferation of hepatocytes in CCl_4_-treated Smad4^Δhep^ mice was significantly decreased compared with that in Smad4^fl/fl^ mice (Figure 3A). To further elucidate the role of Smad4 in hepatocytes, we used siRNA to knock down Smad4 in AML-12 cells, and then the cells were treated with TGF-β1 for 12 h and 24 h to simulate liver fibrosis environment in vitro [21]. The proliferation and migration ability of AML-12 cells were detected by MTT and wound-healing assays. We found that Smad4 deletion remarkably inhibited the proliferation and migration of AML-12 cells after 24 h (Figure 3B–D). It has been reported that EMT of hepatocytes can not only partly become the source of myofibroblasts and promote liver fibrosis [7], but also promote the motility of hepatocytes [22]. Therefore, we speculated that Smad4 might play a role in the EMT of hepatocytes. As expected, we observed much higher expression of E-cadherin in the liver tissues of CCl_4_-treated Smad4^Δhep^ mice than that in Smad4^fl/fl^ mice (Figure 3E). Consistently, Western blotting results also confirmed that siRNA-mediated Smad4 knockdown blocked TGF-β1-induced E-cadherin downregulation in AML-12 cells (Figure 3F). Thus, these findings collectively demonstrated that the knockout of Smad4 in hepatocytes suppressed their proliferation and EMT during liver fibrosis.

### 2.4. Hepatocyte-Specific Smad4 Deficiency Reduced ID1 and CTGF Expression

**Figure 4:**
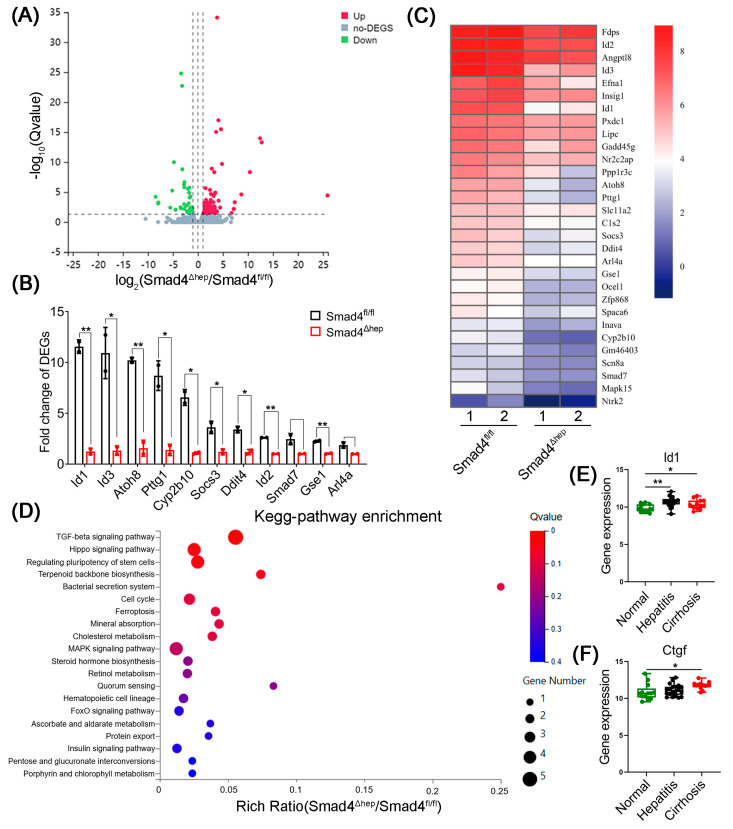
RNA sequencing analysis of DEGs in CCl_4_-induced liver fibrosis tissues. Smad4^fl/fl^ and Smad4^Δhep^ mice were treated with CCl_4_ for 4 weeks to establish the liver fibrosis model. The fibrotic liver tissues of Smad4^fl/fl^ and Smad4^Δhep^ mice were analyzed by RNA sequencing (*n* = 2 per group). (**A**) Volcano diagram of DEGs with *p* < 0.05. (**B**) Analysis of fold change in DEGs. (**C**) Heatmap of the expression of the most significantly downregulated genes. (**D**) The analysis of the related signal pathways of downregulated genes by KEGG. (**E**,**F**) The analysis of ID1 and CTGF expression in liver tissues from patients with hepatitis and cirrhosis in the GSE89377 dataset. Normal, *n* = 13; Hepatitis, *n* = 20; Cirrhosis, *n* = 14. * *p* < 0.05, ** *p* < 0.01.

To elucidate the detailed changes of gene expression between Smad4^Δhep^ and Smad4^fl/fl^ mice, we performed protein-coding mRNA-sequencing analysis of liver tissues derived from CCl_4_-induced liver fibrosis mice. A total of 149 DEGs were identified, including 99 upregulated and 50 downregulated genes (Figure 4A). The top 30 DEGs involved in the occurrence of liver fibrosis were selected and displayed as the heat map, which revealed that ID1 expression was markedly decreased in CCl_4_-treated Smad4^Δhep^ mice compared with that in Smad4^fl/fl^ mice (Figure 4B,C). It was reported that the upregulation of ID1 in hepatocytes was accompanied by the upregulation of CTGF expression [23]. To further verify this result, we analyzed a public GEO dataset (GSE89377) and found that, compared to healthy individuals, the expression of ID1 and CTGF was dramatically increased in the liver tissues of patients with hepatitis and cirrhosis (Figure 4E,F). Therefore, we speculated that ID1 and CTGF might play important roles in liver fibrosis of Smad4^Δhep^ mice and Smad4^fl/fl^ mice.

**Figure 5:**
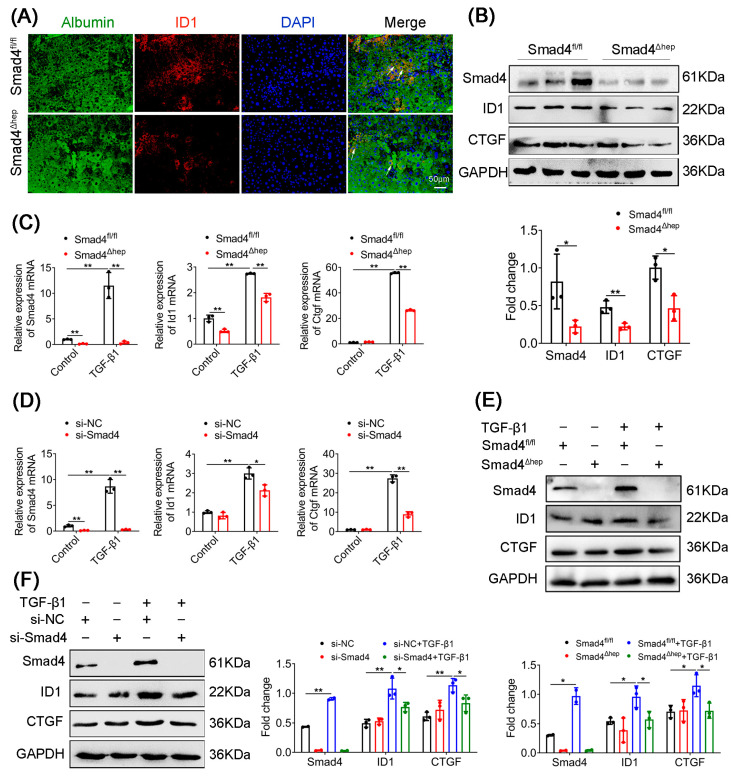
Hepatocyte-specific Smad4 deficiency inhibited the expression of ID1 and CTGF. Smad4^fl/fl^ and Smad4^Δhep^ mice were treated with CCl_4_ for 4 weeks to establish the liver fibrosis model (*n* = 6 per group). Primary hepatocytes were isolated from Smad4^fl/fl^ and Smad4^Δhep^ mice and then treated with TGF-β1 (5 ng/mL) for 24 h. AML-12 cells were transfected with control siRNA or Smad4 siRNA, respectively, and then treated with TGF-β1 (5 ng/mL) for 24 h. (**A**) Double staining of Albumin (green) and ID1 (red) in fibrotic liver tissues (scale bars: 50 μm) and statistical analysis. Arrowhead indicated the double-positive cells. (**B**) Western blotting analysis of protein levels of Smad4, ID1, and CTGF in fibrotic liver tissues. Smad4, ID1, and CTGF were normalized to GAPDH. (**C**) The mRNA levels of Smad4, ID1, and CTGF in primary hepatocytes were measured using real-time PCR analysis. (**D**) The mRNA levels of Smad4, ID1, and CTGF in AML-12 cells were measured using real-time PCR analysis. (**E**) Western blot analysis of protein levels of Smad4, ID1, and CTGF in primary hepatocytes. Smad4, ID1, and CTGF were normalized to GAPDH. (**F**) Western blot analysis of protein levels of Smad4, ID1, and CTGF in AML-12 cells. Smad4, ID1, and CTGF were normalized to GAPDH. * *p* < 0.05, ** *p* < 0.01.

To further explore the interaction between Smad4 and ID1, we detected the expression of Albumin and ID1 in the liver tissues of Smad4^Δhep^ mice using double immunostaining. As shown in Figure 5A, a lower expression of ID1 was observed in Albumin^+^ hepatocytes from CCl_4_-treated Smad4^Δhep^ mice. Consistently, Western blotting results confirmed that the expression of ID1 and CTGF was dramatically downregulated in the liver tissues of Smad4^Δhep^ mice compared with those in Smad4^fl/fl^ mice (Figure 5B). To further verify the above results, we isolated primary hepatocytes from Smad4^Δhep^ mice and Smad4^fl/fl^ mice, respectively, and used si-Smad4 to knockdown Smad4 in AML-12 cells, followed by TGF-β1 stimulation. qRT-PCR and Western blotting analysis revealed that the expression of ID1and CTGF in primary hepatocytes and AML-12 cells was markedly reduced after Smad4 knockout (Figure 5C–F), consistent with in vivo results. Collectively, these results indicated that Smad4 deficiency in hepatocytes decreased the expression of ID1 and CTGF, which may be involved in the process of liver fibrosis.

### 2.5. CTGF Promotes HSCs Activation via p38/p65 Signaling

**Figure 6:**
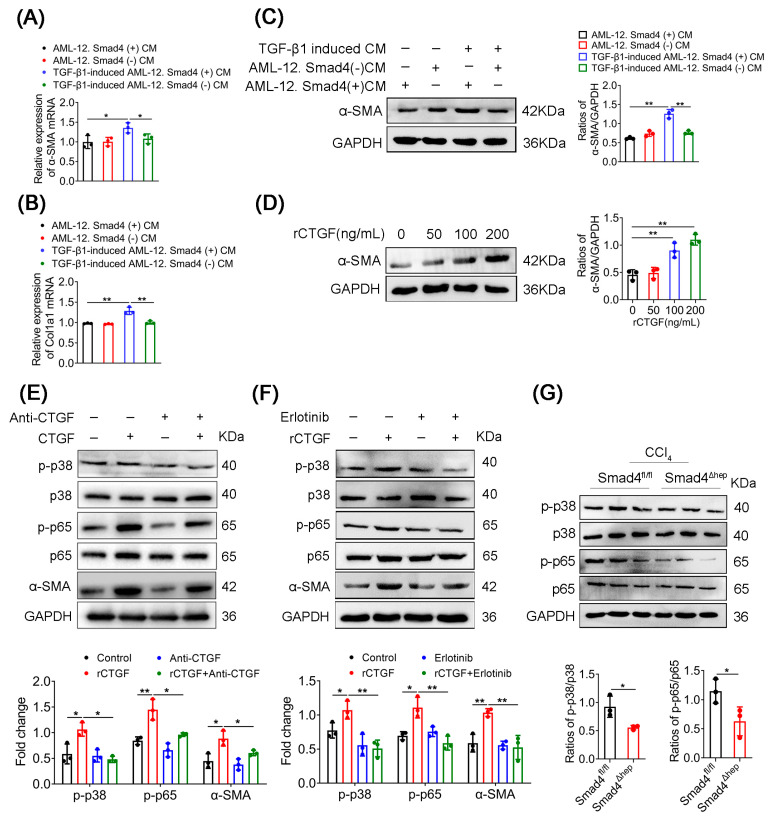
CTGF promotes HSCs activation via the EGFR receptor mediated p38/p65 signaling. AML-12 cells were transfected with control siRNA or Smad4 siRNA, respectively, and treated with TGF-β1 (5 ng/mL) for 24 h. Subsequently, the conditioned medium was collected to treat LX-2 cells. (**A**,**B**) The mRNA levels of α-SMA and Col1a1 in LX-2 cells were measured using the real-time PCR method. (**C**) Western blotting analysis of α-SMA protein levels in LX-2 cells. α-SMA was normalized to GAPDH. (**D**) LX-2 cells were treated with recombinant CTGF protein (0 ng/mL, 50 ng/mL, 100 ng/mL, 200 ng/mL) for 48 h. Western blotting analysis of protein levels of α-SMA in LX-2 cells. α-SMA was normalized to GAPDH. (**E**) LX-2 cells were treated with 200 ng/mL recombinant CTGF and 10 ug/mL CTGF-neutralizing antibody (Anti-CTGF) for 48 h. Western blotting analysis of protein levels of p-p38, p38, p-p65, p65, and α-SMA in LX-2 cells. p-p38 and p-p65 were normalized to p38 and p65, respectively, and α-SMA was normalized to GAPDH. (**F**) LX-2 cells were treated with 200 ng/mL recombinant CTGF and 10 nM Erlotinib for 48 h. Western blotting analysis of protein levels of p-p38, p38, p-p65, p65, and α-SMA in LX-2 cells. p-p38 and p-p65 were normalized to p38 and p65, respectively, and α-SMA was normalized to GAPDH. (**G**) Western blotting analysis of protein levels of p-p38, p38, p-p65, and p65 in fibrotic liver tissues. p-p38 and p-p65 were normalized to p38 and p65, respectively. * *p* < 0.05, ** *p* < 0.01.

Since HSCs activation is a major event in the pathogenesis of liver fibrosis [1], we further explored the underlying mechanism by which Smad4 expression in hepatocytes affected the activation of HSCs. First, AML-12 cells were treated with si-Smad4 and si-NC, respectively, followed by stimulation with TGF-β1. Their culture supernatants were collected as conditioned medium (CM). HSCs were incubated with the above CM for 24 h. In the TGF-β1-free CM treatment groups, the expression of α-SMA and Col1a1 in HSCs was not significantly affected by the absence of Smad4 (Figure 6A,B). Although the CM of TGF-β1-induced AML-12 cells activated the expression of α-SMA and Col1a1 in HSCs, the CM of TGF-β1-induced AML-12 cells with Smad4 knockdown significantly attenuated the expression of α-SMA in HSCs, which was consistent with the results of Western blotting analysis (Figure 6C). During liver fibrosis, both hepatocytes in damaged liver and hepatocytes cultured in vitro express a large amount of CTGF, which increases the pro-fibrotic effect of TGF-β [24]. To investigate whether Smad4 in hepatocytes can promote the activation of HSCs through regulating CTGF, LX-2 cells were treated with exogenous recombinant protein CTGF (rCTGF) at different concentrations for 24 h. Interestingly, the results indicated that the expression level of α-SMA significantly increased in a concentration-dependent manner in LX-2 cells after rCTGF treatment (Figure 6D). To further confirm our findings, we stimulated LX-2 cells with rCTGF and a CTGF-neutralizing antibody, and the results showed that the presence of the CTGF-neutralizing antibody rescued CTGF-mediated HSCs activation (Figure 6E). Recent studies have reported that CTGF plays an important role in renal fibrosis by binding to EGFR on the cell surface [25]; we speculate that CTGF may activate HSCs in an EGFR-dependent manner. Therefore, we treated LX-2 cells with rCTGF and the EGFR-specific inhibitor (Erlotinib) and found that Erlotinib obviously inhibited CTGF-mediated HSCs activation (Figure 6F).

To determine the molecular mechanism via which hepatocyte-derived CTGF acts on HSCs to promote fibrosis, we further analyzed the RNA sequencing results and found that the proteins correlated with MAPK signaling pathway were markedly downregulated in Smad4^Δhep^ mice (Figure 4D). The p38-MAPK and p65-NF-κB pathways have been reported to play key roles in the process of liver fibrosis [26,27]. Consistently, we found that the expression of phosphorylated p38 (p-p38) and p65 (p-p65) in the liver tissue of CCl_4_-treated Smad4^Δhep^ mice was lower than that in Smad4^fl/fl^ mice (Figure 6G). In addition, after LX-2 cells were treated with rCTGF, the phosphorylation of p38 and p65 were distinctly enhanced, whereas both CTGF-neutralizing antibody and erlotinib suppressed this effect (Figure 6E,F). Taken together, these results suggested that CTGF promoted HSCs activation through EGFR receptor-mediated p38 and p65 pathways during liver fibrosis.

## 3. Discussion

**Figure 7:**
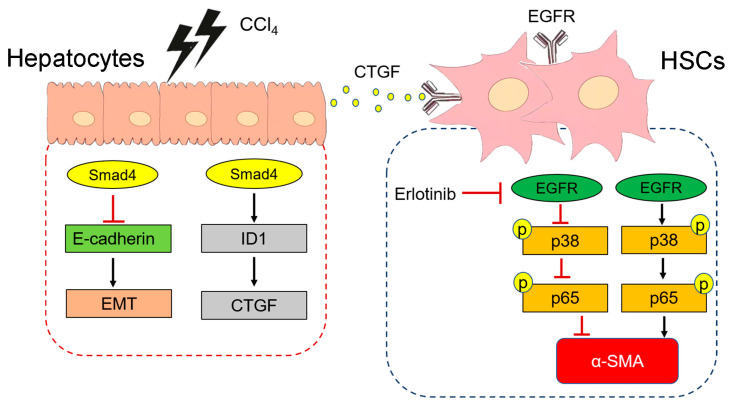
Schematic diagram of the mechanism via which hepatocyte-specific Smad4 deficiency alleviates liver fibrosis. In liver fibrosis, the expression of Smad4 in hepatocytes was upregulated, leading to increased ID1 and CTGF expression in hepatocyte; thereafter, secreted CTGF upregulated the phosphorylation of p38 and p65 via the EGFR receptor to promote HSC activation and liver fibrosis.

Smad4 is a core mediator of the TGF-β signaling pathway that can interact with Smad2/3 to transmit upstream Smad signals and promote the occurrence and development of liver fibrosis [12]. Studies have shown that some drugs, such as Ferulic acid [28], Yu Gan Long [29], and Magnesium Isoglycyrrhizinate [30], can inhibit the expression of Smad4 in TGF-β signaling pathway, thereby inhibiting liver fibrosis. However, the specific contribution of hepatocyte Smad4 during liver fibrosis remains unclear. Here, we used a mouse model of hepatocyte-specific Smad4 deletion to explore its role and molecular mechanism in liver fibrosis. Notably, we found that hepatocyte-specific Smad4 deletion alleviated CCl_4_-induced liver fibrosis and suppressed hepatocyte proliferation and EMT. Furthermore, Smad4 was able to regulate ID1 expression and CTGF secretion in hepatocytes to activate the p38 and p65 signaling pathways in HSCs and thereby promote HSCs’ activation (Figure 7).

Accumulating evidence has shown that the dysregulation of the TGF-β1/Smad pathway is a major contributor in the pathogenesis of liver inflammation, fibrosis, and HCC. Thus, the imbalance of Smad signal plays an important role in the development of liver fibrosis [31]. Additionally, studies have reported that Smad4-mediated signal transduction in different cell types plays different roles in liver fibrosis. For instance, some studies have reported that Smad4 deficiency in hepatocytes does not affect liver development, but gradually results in iron overload and the infiltration of inflammatory cells in the liver and other organs of mice [19]. However, others demonstrated that Smad4 deletion in HSCs attenuated their activation and reduced the expression of pro-fibrotic genes [18]. Qin et al. found that the expression of inflammatory markers, fibrotic markers, and lipogenic genes was significantly lower in the liver tissue of hepatocyte-specific Smad4-deficient NASH mice than that in wild-type mice [16]. Yang et al. confirmed that Smad4 deletion in hepatocytes after knocking out *TAK1* inhibited the apoptosis of hepatocytes and decreased serum ALT levels, while it simultaneously alleviated liver inflammation, fibrosis, and HCC [17]. Xu et al. reported that the expression of fibrotic genes, such as TIMP1 and TGF-β, in Smad4-knockout mice was dramatically lower than that in WT mice, suggesting that the TGF-β1/Smad signal transduction system was downregulated [32]. Together, our findings support this conclusion and demonstrate that hepatocyte-specific Smad4 deletion reduces CCl_4_-induced liver fibrosis.

Hepatocytes, as the most abundant parenchymal cells in the liver, are the initial cells that affect the process of liver fibrosis. During serious liver injury, hepatocytes lose the ability of regeneration and undergo necrosis, apoptosis, or senescence, while activated myofibroblasts in the liver to secrete ECM proteins [33]. Hepatocytes can be transformed into myofibroblasts through EMT, which is an important source of myofibroblasts in the process of liver fibrosis [7]. Some studies have shown that inhibiting the EMT of hepatocytes can reduce liver fibrosis [34]. Importantly, Kaimori et al. reported that TGF-β1 could induce EMT in AML-12 cells in vitro, whereas Smad4 knockdown in AML-12 cells inhibited EMT [35]. Consistently, we found that Smad4 deletion in hepatocytes alleviated EMT and preserved the expression of the epithelial marker, E-cadherin, which suggested that the absence of Smad4 in hepatocytes attenuated the development of liver fibrosis. However, Taura et al. also demonstrated that hepatocytes did not undergo EMT during liver fibrosis [36]; therefore, the function and mechanism of EMT in hepatocytes during liver fibrosis still needs to be further explored.

Damaged hepatocytes secrete inflammatory factors (e.g., IL-33 and NLRP3) and fibrotic factors (e.g., TGF-β1 and CTGF) that are involved in HSCs activation and promote liver inflammation and fibrosis [4,37]. In this study, we found that the expression of ID1 and CTGF in hepatocytes was markedly downregulated in hepatocyte-specific Smad4 deletion mice with liver fibrosis. ID1 is mainly correlated with tumorigenesis, cell senescence, cell proliferation, and survival, and is overexpressed in various cancer cells and can promote tumor development through different signaling pathways [38]. Moreover, Young et al. reported that the ID1 mRNA level was significantly upregulated in liver biopsy specimens from chronic hepatitis C patients, and that phosphorylated Smad1/5 and ID1 expression were dramatically enhanced in HCV-infected hepatoma cells [39]. Meanwhile, Yin et al. also found that ID1 deletion inhibited cell proliferation and sensitized oxaliplatin-resistant HCC cells to death [40]. Interestingly, liver-specific Smad4 knockout also markedly weakened ID1 expression [41], which is consistent with our findings.

CTGF is a strongly fibrogenic molecule that is overexpressed in fibrotic organs, including liver, lung, kidney, and heart [37]. Kodama et al. previously demonstrated that p53 overexpression in hepatocytes could promote the expression of CTGF to increase hepatocyte apoptosis and spontaneous liver fibrosis [42]. Similarly, Makino et al. found that upregulated CTGF expression was positively correlated with the clinical malignancy of HCC, and that CTGF-specific knockout in HepG2 reduced the size and number of liver tumors. Thus, CTGF derived from HCC appears to be a key factor in activating nearby HSCs and relaying pro-growth signals to HCC [37]. Additionally, CTGF is also reported to be a downstream mediator of TGF-β, and its expression is enhanced when stimulating hepatocytes with TGF-β [43]. Here, we verified the correlation between ID1, CTGF, hepatitis, and cirrhosis in clinical cases by analyzing the expression of ID1 and CTGF in 20 healthy individuals, 14 hepatitis patients, and 13 cirrhosis patients using the GEO dataset GSE89377. As expected, we found that the expression of ID1 and CTGF was distinctly increased in patients with hepatitis and cirrhosis.

HSCs’ activation is a key step in the development of liver fibrosis. As the main effector cells of the fibrosis response, HSCs are particularly important autocrine or paracrine targets, especially in the activated state [4,43]. Liao et al. demonstrated that ID1 and MAPK signaling pathways were downstream of CTGF signaling, and ID1 partially upregulated CTGF through positive feedback [23]. Here, our results indicated that Smad4 expression in hepatocytes could activate HSCs through improving CTGF secretion, and thereby promoted liver fibrosis. Huang and Brigstock also confirmed that CTGF could promote liver fibrosis by promoting proliferation, survival, migration, adhesion, and ECM production of activated HSCs [44]. This result is consistent with our findings. It was recently reported that CTGF was able to regulate renal inflammation, cell growth, and fibrosis by binding to EGFR [25,45]. Therefore, we speculated that CTGF derived from hepatocytes might stimulate the activation of HSCs via EGFR. Interestingly, our results showed that the presence of erlotinib (EGFR inhibitor) attenuated HSCs’ activation stimulated by CTGF, and that the p38/MAPK signaling pathway was downregulated in CCl_4_-induced liver fibrotic tissues of Smad4^Δhep^ mice. Consistently, Fuchs et al. also found that erlotinib could inhibit the activation of HSCs by reducing EGFR phosphorylation in HSCs [46]. At present, some studies have taken the inhibition of HSCs’ activation as a therapeutic target for liver fibrosis [4]. Yan et al. confirmed that phosphorylated p38 was upregulated in activated HSCs [27]. p38 is known to play an important role in the process of liver fibrosis [23], and the activation of p38α/MAPK promotes hepatocyte proliferation and chronic liver inflammation [47]. Interestingly, Ras/Raf/MAPK and PI3K/AKT, two major signaling pathways downstream of EGFR, can promote mitosis and prevent apoptosis in lung cancer cells [48]. Phosphorylated EGFR can trigger downstream signaling to promote proliferation, metastasis, and angiogenesis of lung cancer cells [49]. As a ubiquitous inducible transcription factor, nuclear factor-κB (NF-κB) can mediate the expression of a large number of intracellular genes involved in differentiation, apoptosis, and proliferation [50,51]. The activation of NF-κB can affect the activation of HSCs and promote liver inflammation, fibrosis, and hepatocellular carcinoma [50,52]. Some studies suggested that p38/MAPK was associated with the inflammatory signaling pathway p65/NK-κB in chronic hepatitis and HCC [26]. Consistently, our study demonstrated that hepatocyte-derived CTGF increased the phosphorylation of p38 and p65 and promoted HSCs’ activation through EGFR, thereby contributing to liver fibrosis.

In conclusion, our research indicated that Smad4 expression in hepatocytes is closely involved in the development of liver fibrosis. Notably, Smad4 deletion in hepatocytes alleviated CCl_4_-induced liver fibrosis and decreased inflammatory cell infiltration in liver tissues. Molecularly, Smad4 expression in hepatocytes upregulated the expression of ID1 and further enhanced the paracrine activity of CTGF; subsequently, mediated by EGFR, CTGF promoted HSCs’ activation by regulating the p38 and p65 signaling pathways, which in turn led to liver fibrosis. However, we used CCl_4_ to induce short-term fibrosis, and the functional role of hepatocyte Smad4 in long-term liver fibrosis needs to be further studied. Collectively, Smad4 may represent a potential candidate target for the prevention and targeted therapy in liver fibrosis.

## 4. Materials and Methods

### 4.1. Animals

Albumin-Cre (Alb-Cre) and Smad4^flox/flox^ (Smad4^fl/fl^) mice on a C57BL/6 background were purchased from Jackson Laboratory (Bar Harbor, ME, USA) [53]. Mice with a conditional Smad4 knockout in Alb-expressing hepatocytes (Smad4^Δhep^) were generated by crossing Smad4^fl/fl^ and Alb-Cre mice. All mice genotypes were verified by PCR for three times before subsequent experiments. Then, 8- to 10-week-old male Smad4^Δhep^ mice and control littermate mice were used for the experiments. All animal experiments were performed after being approved by the Institutional Laboratory Animal Care and Use Committee of College of Science, Beijing Jiaotong University. All mice were housed under specific pathogen-free conditions with a 12 h light/dark cycles in humidity of 40-70% and at an ambient temperature of 18-26 °C. Mice were fed regularly with diet pellets and had free access to water.

### 4.2. Carbon Tetrachloride (CCl_4_)-Induced Acute Liver Fibrosis Model

To induce acute liver fibrosis, mice were injected intraperitoneally with CCl_4_ mixed with corn oil (1:9, Sigma-Aldrich, St. Louis, MO, USA) at a dose of 0.5 μL CCl_4_/g body weight twice weekly for 4 weeks, and control mice were injected intraperitoneally with the same dose of corn oil [2]. Twenty-four hours after the final CCl_4_ injection, mice were sacrificed and their tissues were harvested.

### 4.3. Immunohistochemistry and Immunofluorescence Analysis

Paraffin-embedded and frozen sections of liver tissues were prepared as described previously [2]. For immunohistochemical analysis, paraffin-embedded sections were stained with hematoxylin and eosin (H&E) and Sirius Red, respectively. For immunofluorescence detection, paraffin sections were incubated with anti-Albumin (Affinity Biosciences, Cincinnati, OH, USA), anti-Smad4 (Santa Cruz Biotechnology, Shanghai, China), anti-α-SMA (Abcam, Cambridge, UK), anti-collagen I (Affinity Biosciences, Cincinnati, OH, USA), and anti-PCNA (Santa Cruz Biotechnology, Shanghai, China) primary antibodies, respectively; frozen sections were incubated with anti-F4/80, anti-CD11b, and anti-Gr-1 primary antibodies (BD Pharmingen, San Diego, CA, USA), respectively, and followed by incubation with Alexa Fluor 488- or 594-conjugated secondary antibodies (Invitrogen, Carlsbad, CA, USA). Cell nuclei were stained with DAPI. The results were evaluated under the microscope (DP71, OLYMPUS, Tokyo, Japan). Image J software (Image J 1.8.0, NIH, Bethesda, USA) was used to quantify the collagen deposition in Sirius Red staining and the positive areas in immunohistochemistry and immunofluorescence, which are presented in the form of percentage.

### 4.4. Western Blotting Analysis

Western blotting was performed as described previously [54]. Briefly, cells and liver tissue samples were collected and lysed in RIPA buffer supplemented with a protease inhibitor cocktail (Solarbio, Beijing, China). Protein concentration was measured using a BCA protein assay kit (LABLEAD, Beijing, China). Proteins were separated by 10% SDS-PAGE gel at 115 V for 1.2 h, then were transferred to a PVDF membrane at 200 mA for 1 h. The membranes were blocked with 5% milk in TBST for 1 h and incubated overnight at 4 °C with primary antibodies. Primary antibodies included anti-Smad4, anti-α-SMA, anti-GAPDH, anti-E-cadherin, anti-ID1, anti-CTGF, anti-p65, anti-p-p65, anti-p38, and anti-p-p38 (Affinity Biosciences, Cincinnati, OH, USA). Followed by HRP-conjugated goat, anti-mouse and goat anti-rabbit IgG (Solarbio, Beijing, China) were used as secondary antibodies. Protein bands were scanned using a Clinx Science Instrument and quantified with Image J software.

### 4.5. Isolation of Mouse Primary Hepatocytes

Primary mouse hepatocytes were isolated using a two-step collagenase digestion and gradient centrifugation method, as described previously [55]. Filtered cells were centrifuged at 50× *g* for 3 min to collect hepatocytes, which were then resuspended in 10 mL DMEM (HyClone, Logan, UT, USA) and placed on top of 40% percoll and centrifuged at 800× *g* for 10 min. The hepatocyte fraction at the bottom of the layers was collected, and cell viability was examined by Trypan blue exclusion. Both the cell purity and viability were greater than 90%. Mouse primary hepatocytes were treated with 5 ng/mL TGF-β1 (Sino Biological, Beijing, China) for 24 h.

### 4.6. Cell Culture

Mouse primary hepatocytes were cultured in William’s E medium (Gibco, Grand Island US) supplemented with 10% fetal bovine serum (FBS, BI, Kibbutz Beit Haemek, Israel) and 1% penicillin/streptomycin. AML-12 hepatocyte cells (ATCC, Manassas, VA, USA) were cultured in DMEM/F12 medium (BI, Israel) supplemented with 10% FBS, 1% penicillin/streptomycin, 1% insulin-transferrin-selenium (ITS, Procell, Wuhan, China), and 40 ng/mL dexamethasone (Solarbio, Beijing, China). The human HSC LX-2 cell line was purchased from Xiangya Medical Collage (Changsha, China). LX−2 cells were cultured in DMEM supplemented with 10% FBS and 1% penicillin/streptomycin at 37 °C with 5% CO_2_. AML-12 cells were treated with 5 ng/mL TGF-β1 (Sino Biological, Beijing, China) for 24 h. LX-2 cells were treated with 200 ng/mL CTGF recombinant protein (rCTGF, Cloud-Clone Corp, Wuhan, China), 10 ug/mL CTGF-neutralizing antibody (Pepro Tech, NJ, USA), or 10 μM Erlotinib (MedChemExpress, Princeton, NJ, USA) for 48 h.

### 4.7. Small Interfering RNA (siRNA) Interference

Smad4-targeting siRNA (si-Smad4) and control siRNA (si-NC) were purchased from GenePharma (Suzhou, China). AML-12 cells were transfected with 53.3 nM siRNA using si-mate transfection reagent (GenePharma, Suzhou, China) according to the manufacturer’s protocols.

### 4.8. Quantitative Real-Time Polymerase Chain Reaction (qPCR)

Total RNA was isolated from liver tissues and cells using TRIzol reagent (Invitrogen, Carlsbad, CA, USA) according to the manufacturer’s instructions. cDNA was synthesized using a Primescript RT Master Mix Kit (MedChemExpress, Princeton, NJ, USA). qPCR was performed in duplicate with a SYBR Premix Ex TaqTM Kit (MedChemExpress, Princeton, NJ, USA). Data were analyzed using the 2^−ΔΔCt^ method and normalized to GAPDH expression.

### 4.9. Cell Viability Analysis

AML-12 cells were cultured in a 96-well plate at a density of 5 × 10^3^ cells per well and then transfected with si-Smad4 and stimulated with 5 ng/mL TGF-β1 after 48 h. The viability was detected by 3-(4,5-dimethylthiazol-2-yl)-2,5-diphenyl-tetrazolium bromide (MTT) method according to the manufacturer’s protocols. The OD value of cells was analyzed at 12 h and 24 h, respectively.

### 4.10. Wound-Healing Assay

AML-12 cells were cultured in 6-well plate and transfected with si-Smad4 and allowed to grow until confluent. The cell layer was scratched with a 200 μL pipette tip. After scratching, cells were washed with serum-free medium and incubated in complete DMEM/F12 media with 5 ng/mL TGF-β1. The scratch areas were photographed at 0 h and 24 h, respectively. Quantification of wound healing was performed using Image J software.

### 4.11. Flow Cytometry Analysis

Flow cytometry was performed as described previously [56]. Single-cell suspensions were collected from liver tissues and spleen tissues and incubated with the following directly labeled mouse-specific monoclonal antibodies: FITC-labeled anti Gr1, APC-labeled anti F4/80, Percp-labeled anti CD11b, and APC-labeled anti Gr1. Cells were collected on a FACSCalibur (BD Biosciences, San Diego, CA, USA) and analyzed by FlowJo software (TreeStar, Ashland, OR, USA).

### 4.12. RNA Sequencing Analysis

To explore potential genes involved in liver fibrosis, RNA sequencing analysis was performed as described previously [57]. The total RNA of liver fibrosis tissues from Smad4^fl/fl^ and Smad4^Δhep^ mice (*n* = 2 per group) was extracted with RNeasy Mini Kit (QIAGEN, Dusseldorf, Germany). RNA-sequencing analysis was performed using the BGISEQ-500 sequencer platform by BGI (Shenzhen, China). Differentially expressed genes (DEGs) were identified with a *p* value of <0.01, and an absolute log2 Ratio of ≥1. The Kyoto encyclopedia of Genes and Genomes (KEGG) enrichment analysis was performed by using Phyper in R. All analyses were conducted on the Dr Tom network platform of BGI (http://report.bgi.com) (accessed on 3 March 2021).

### 4.13. Public Database Analysis

The expression of ID1 and CTGF in clinical samples from hepatitis, cirrhosis patients, and healthy individuals was analyzed using raw gene expression data (GSE89377) [58], downloaded from the Gene Expression Omnibus (http://www.ncbi.nlm.nih.gov/geo) (accessed on 8 April 2021).

### 4.14. Statistical Analysis

All data are shown as the mean ± SD and were analyzed using GraphPad Prism software. Significant differences between mean values were obtained using three independent experiments. Differences between the two groups were compared using two-tailed unpaired Student’s t-test analysis. One-way ANOVA tests with a Bonferroni correction were used for multiple comparisons. A statistically significant value was set at *p* < 0.05.

## Data Availability

All relevant data are presented in this paper.

## Supplementary Materials

The following supporting information can be downloaded at: https://www.mdpi.com/article/10.3390/ijms231911696/s1.
