# Supplementary material for: Hepatocyte-Specific Smad4 Deficiency Alleviates Liver Fibrosis via the p38/p65 Pathway
Source: Int J Mol Sci. 2022 Oct 2;23(19):11696. doi: 10.3390/ijms231911696 (PMC9570188; doi:10.3390/ijms231911696)
Supplement: Supplementary file 1 [file ijms-23-11696-s001.zip › ijms-1844266-supplementary.pdf]

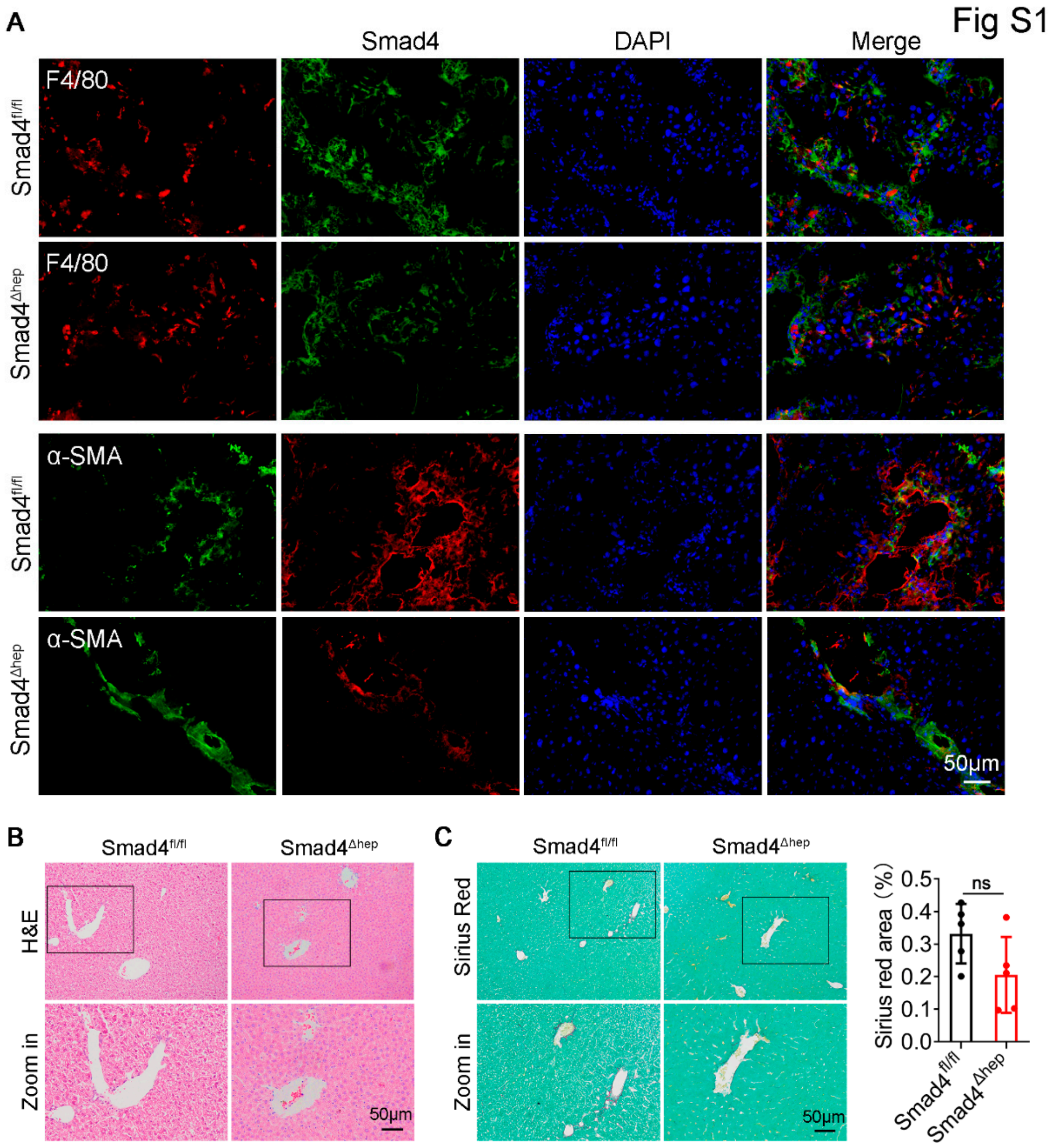

Figure S1. Detection of Smad4-specific deletion in hepatocytes of Smad4Δhep and Smad4fl/fl mice.

(A) Representative immunofluorescence analysis of Smad4 expression in macrophages and myofibroblasts from CCl_4-treated Smad4$^{\Delta hep}$ and Smad4$^{fl/fl}$ mice. F4/80 and *α*-SMA were used as cell-specific markers (scale bar: 50μm). (B) H&E staining of liver tissues from Smad4$^{\Delta hep}$ and Smad4$^{fl/fl}$ mice (scale bars: 100μm, zoom in: 50μm). (C) Sirius Red staining of liver tissues from Smad4$^{\Delta hep}$ and Smad4$^{fl/fl}$ mice (scale bars: 100μm, zoom in: 50μm). *p <0.05, **p <0.01.

Fig S2

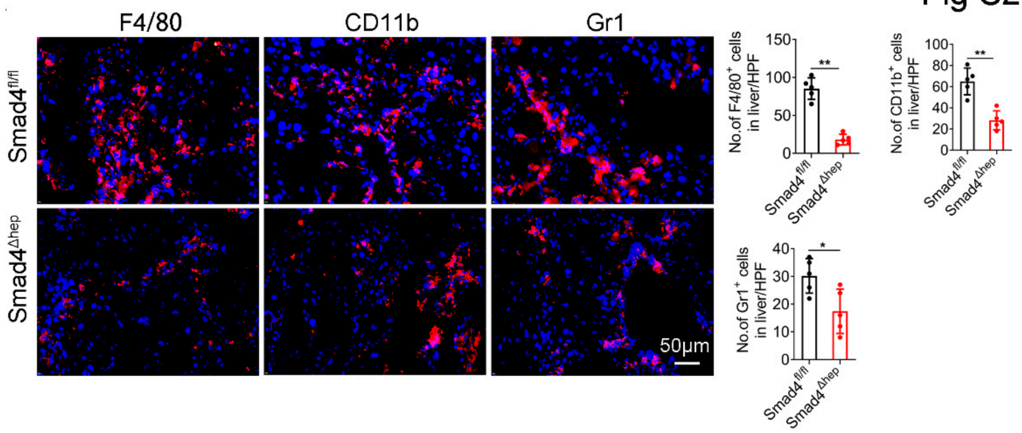

Figure S2. Smad4 deficiency in hepatocytes attenuates liver inflammatory response. Groups of Smad4$^{fl/fl}$ and Smad4$^{\Delta hep}$ mice (n = 6 per group) were treated with CCl_4 for 4 weeks to establish the liver fibrosis model. Representative staining and statistical analysis of F4/80, CD11b and Gr1 in liver tissues (scale bar: 50μm). *p <0.05, **p <0.01.
